# Effects of Total Intravenous Anesthesia and Low- and High-Flow Anesthesia Implementation on Middle Ear Pressure

**DOI:** 10.1155/2018/8214651

**Published:** 2018-04-10

**Authors:** Ayca Dumanli Ozcan, Aysun Ersen Yungul, Togay Muderris, Hulya Kasikara, Helin Sahinturk, Orhan Kanbak, Abdulkadir But

**Affiliations:** ^1^Department of Anesthesiology and Reanimation, Ankara Atatürk Training and Research Hospital, Ankara, Turkey; ^2^Department of Otorhinolaryngology, Ankara Atatürk Training and Research Hospital, Ankara, Turkey; ^3^Department of Anesthesiology and Reanimation, Yıldırım Beyazıt University, Ankara, Turkey

## Abstract

**Background:**

The middle ear is an air-filled lacuna in the temporal bone. Inhaled anesthetic agents increase the pressure of this lacuna. Therefore, attention must be paid in choosing not only anesthetic agents but also anesthetic method.

**Aim:**

This study compared the effects of high-flow total intravenous anesthesia (TIVA) and low- and high-flow desflurane anesthesia on middle ear pressure.

**Study Design:**

Randomized prospective double-blind study.

**Methods:**

In this retrospective double-blind study, 90 patients (20–65 years old) scheduled to undergo elective thyroidectomies were divided into three randomized anesthesia groups: high-flow desflurane (Group I), low-flow desflurane (Group II), and high-flow TIVA (propofol, remifentanil) (Group III). The hemodynamic and respiratory parameters and tympanometry were measured before induction (*T*1), 10 minutes after intubation (*T*2), 10 minutes before the end of the operation (*T*3), and 5 (*T*4), 10 (*T*5), 15 (*T*6), and 30 (*T*7) minutes after the operation.

**Results:**

No statistically significant differences were found in the age, gender, weight, height, body mass index, surgery duration, and anesthetic duration (*p* > 0.05). There were no statistically significant differences at *T*1, *T*3, *T*4, *T*5, *T*6, and *T*7 (*p* > 0.007), but there was a significant difference at *T*2 (*p* < 0.001), with Groups II and III having lower pressure than Group I (*p* < 0.001).

**Conclusion:**

The high-flow desflurane group had higher postinduction middle ear pressure values. Therefore, low-flow anesthesia and TIVA can be used more safely in middle ear surgeries, provided that a well-equipped anesthetic device and appropriate monitoring conditions are available.

## 1. Introduction

The middle ear is an air-filled lacuna, with a volume of approximately 0.5 cm, located in the temporal bone. Inhaled anesthetic agents increase the pressure of this lacuna. Therefore, attention must be paid in choosing anesthetic agents that cause minimal intratympanic pressure increases to prevent adverse effects [1, 2], including middle ear condition changes, haemotympanum, serous otitis, temporary or permanent hearing loss, tympanic membrane graft dislocation, or deformation of the ossicular chain.

Nitrous oxide has been demonstrated to cause a time-related increase in pressure with accumulation in a closed environment, which is true for the middle ear [3, 4]. Most previous investigations of the middle ear pressure (MEP) have been performed with nitrous oxide, halothane, sevoflurane, desflurane, and total intravenous anesthesia (TIVA) with propofol [5, 6]. Although there are many benefits of using low-flow anesthesia, such as cost reduction, prevention of environmental pollution, increased humidity of the gases, decreased heat loss, and better preservation of the tracheal and bronchial physiology [7, 8]; its effects on MEP have not been sufficiently researched.

The goal of this study was to investigate the effects of low- and high-flow desflurane and high-flow TIVA (propofol remifentanil) on the MEP in patients undergoing elective thyroidectomies.

## 2. Methods

Following the approval of the ethics committee, 90 American Society of Anesthesiology (ASA) classification I-II patients scheduled for elective thyroidectomies provided informed consent and were enrolled in this study. Those patients with uncontrolled hypertension, active and severe renal failure, hepatic, respiratory, or cardiac disease, neurological disorders, neuromuscular disorders, adenotonsillar hypertrophy, nasal septal deviation, orthoscopic pathologies, the absence of an acoustic reflex, or a flat tympanogram were excluded from this study.

Before the induction of anesthesia, the groups were determined randomly by drawing lots from an envelope containing sheets of paper with the group names. The heart rate (HR), systolic arterial pressure (SAP), diastolic arterial pressure (DAP), mean arterial pressure (MAP), and the peripheral O_2_ saturation (SpO_2_) were monitored during surgery. Intravenous (iv) access was established through a 20-gauge granule, and an infusion of 5–10 ml/kg/h of 0.9% sodium chloride (NaCl) was started. The patients were given 1 mg of lidocaine (2% Animal; Osel, Istanbul, Turkey), 1 mcg/kg of remifentanil (5 mg Ultiva; GlaxoSmithKline, Istanbul, Turkey), 5 mg/kg of thiopental (pental sodium; IE Ulagay), and 0.6 mg/kg of rocuronium (Curon; Mustafa Nevzat, Istanbul, Turkey) iv for the induction of anesthesia. After providing preoxygenation with 100% O_2_ for 3 minutes using a face mask, the patients were intubated when sufficient muscle relaxation was observed. Each patient was ventilated (Dräger, Lübeck, Germany) with a tidal volume of 10 ml/kg and a frequency of 12 breaths/min. Soda-lime (Sorbo-lime, Berkim, Turkey) was used as the CO_2_ absorbent.

For the maintenance of the anesthesia, the patients were randomly divided into 3 groups. In the first 2 groups, the patients were provided with 6% desflurane (Forane; Abbott Laboratories, Queenborough, England), along with 40% O_2_ and 60% air. In Group III (TIVA group), the patients were given 100 *μ*g/kg/min of propofol and 0.25 *μ*g/kg/min of remifentanil, along with 40% O_2_ and 60% air. In Group I, high-flow desflurane was sustained for the first 10 minutes, and when the 6 l/min flow was started, the desflurane was sustained. In Group II (low-flow desflurane), the flow was reduced to 1 l/min after the 6 l/min flow was started for the first 10 minutes. In Group III (high-flow TIVA), a 6 l/min flow was started for the first 10 minutes and maintained.

Ten minutes before the end of the surgery in all of the groups, the flow was changed to 6 l/min and the anesthetic gases were cut off, with 100% oxygen being maintained. Decurarization was ensured in all of the patients with 0.5 mg of atropine and 1.5 mg of neostigmine. The remifentanil infusion doses were adjusted to achieve a 55–60 mmHg mean arterial pressure in the TIVA group. In all of the groups, when the HR fell below 40, 0.5 mg of atropine was administered; when the MAP was below 50, 10 mg of ephedrine was administered and the infusion dose was decreased. Thirty minutes before the end of the operation, the patients were given intravenously 1 mg/kg of tramadol and 10 mg of metoclopramide.

The haemodynamic and respiratory parameters (SAP, DAP, MAP, HR, SpO_2_, and EtCO_2_) were recorded before and after induction, after the intubation, at the beginning of the 6 l/min ventilation, at the beginning of the 1 l/min ventilation, at minutes 5, 10, 15, 30, 60, and 90 of the 1 l/min flow, at the end of the 1 l/min flow, and during extubation. The MEPs were measured via tympanometry (OTOflex 100; Otometrics, Denmark) before induction (*T*1), 10 minutes after the intubation (*T*2), at the end of the 1 l/min ventilation, 10 minutes before the end of the operation (*T*3), and 5 (*T*4), 10 (*T*5), 15 (*T*6), and 30 (*T*7) minutes after the operation.

### 2.1. Statistical Analysis

The data analysis was performed by using SPSS for Windows, version 11.5 (SPSS Inc., Chicago, IL, United States). Whether or not the distributions of the continuous variables were normal was determined via the Kolmogorov-Smirnov test. Levene's test was used for the evaluation of the homogeneity of the variances, and the data are shown as the mean ± SD or median (min–max), where applicable.

The mean differences among the groups were analyzed using the one-way ANOVA, and the Kruskal-Wallis test was applied to compare the medians. The *p* values from the one-way ANOVA post hoc Tukey HSD test were used to determine the differences between the groups. The nominal data were analyzed with Pearson's chi-squared test.

The differences among the repeated measurements were analyzed by a repeated-measures ANOVA. The Bonferroni adjusted multiple comparisons test was used to determine the time measurement differences when the *p* value from the repeated-measures ANOVA was statistically significant. Overall, a *p* value of less than 0.05 was considered to be statistically significant. However, for all possible multiple comparisons, the Bonferroni correction was applied to control Type I errors.

## 3. Results

No statistically significant differences were found among the groups in terms of the clinical and demographic features, such as age, gender, weight, height, body mass index (BMI), and surgical and anesthetic durations (*p* > 0.05) (Table 1). In addition, there were no statistically significant differences among the groups according to the Bonferroni adjustment regarding the percentage changes in the SAP, DAP, MAP, HR, SpO_2_, and EtCO_2_ levels at *T*1, *T*2, *T*3, *T*4, and *T*7, when compared to *T*0 (*p* > 0.0033).

**Figure 1:**
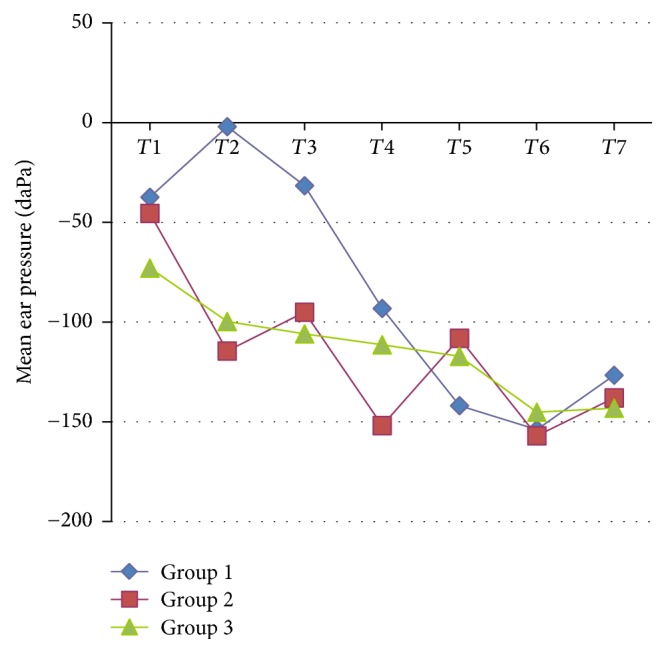
MEP values of the groups at times *T*1,* T*2,* T*3,* T*4,* T*5,* T*6, and* T*7.

No statistically significant differences were found among the groups in terms of the measurements conducted through the ear at *T*1, *T*3, *T*4, *T*5, *T*6, and *T*7 (*p* > 0.007). However, at *T*2, there was a statistically significant difference among the groups (*p* < 0.001), with Groups II and III having lower pressure levels than Group I (*p* < 0.001) (Table 2) (Figure 1).

In Group I, statistically significant decreases in the MEP were noted at *T*5, *T*6, and *T*7 when compared to *T*1; at *T*4, *T*5, *T*6, and *T*7, when compared to *T*2; and at *T*5, *T*6 and *T*7, when compared to *T*3 (*p* < 0.017). In Group II, statistically significant decreases in the MEP were noted at *T*2, *T*4, *T*6, and *T*7, when compared to *T*1, and at *T*4 and *T*6, when compared to *T*3 (*p* < 0.017). In Group III, statistically significant decreases in the MEP were noted at *T*6 and *T*7, when compared to *T*1 (*p* < 0.017).

No statistically significant differences were found among the groups in terms of the measurements conducted through the ear at *T*2, *T*3, *T*4, *T*5, *T*6, and *T*7, when compared to *T*1 (*p* < 0.0024) (Table 3). Moreover, there were no statistically significant differences among the groups according to the Bonferroni correction with regard to the percentage changes in the measurements conducted through ear at *T*4, *T*5, *T*6, and *T*7, when compared to *T*3 (*p* > 0.0024) (Table 4).

## 4. Discussion

In this study, no statistically significant differences were seen with regard to the changes in the hemodynamic parameters, when compared to the initial values. The postinduction middle ear pressure values of the group that received the high-flow desflurane (Group I) were higher than in those who received TIVA and low-flow desflurane. The only group, which had increased intraoperative pressure values, when compared to the initial values, was Group I. The patients that received low-flow anesthesia (Group II) yielded lower intraoperative measures after the induction and before the extubation when compared to the preinduction value (*T*1). In Group II, the pressure values following extubation were also lower than those measured during the intraoperative period. However, no statistically significant changes were seen in the intraoperative MEP values of the TIVA group (Group III). As such, the TIVA group was the most stable in terms of the MEP values, while Group I had the highest increase in the intraoperative ear pressure.

The effects of several agents on the MEP have been investigated in many studies, using nitrous oxide, halothane, sevoflurane, desflurane, isoflurane, and TIVA anesthesia [5, 6]. For example, Acar et al. [9] studied the effects of two different agents on the MEP and found that desflurane increased the intraoperative intratympanic pressure, supporting the findings of our study. In addition, they reported that isoflurane can be used more safely in middle ear operations. Theoretically, desflurane increases the pressure by accumulating in potential spaces due to its low solubility. They demonstrated that isoflurane affects the MEP less, depending on the blood partition coefficients. In addition, Ozturk et al. studied high-flow desflurane anesthesia and did not recommend its use due to the complications arising from the increase in the MEP values [5].

TIVA is the preferred anesthesia since the induction is fast, and the medications used can affect specific receptor regions. The effects are limited, and the dose-response relationship is predictable. It sensitizes the heart against catecholamines and provides better cardiovascular stability. Arrhythmias and myocardial depression occur rarely, and the emergence is faster, making it an advantageous anesthetic method. In their study, Güler et al. [10] recommended TIVA for laparoscopic surgery, since the pressures followed a low course in the TIVA group. They reported that sevoflurane did not increase the ear pressure above 50 daPa and that there were no inconveniences related to its use, but optimum anesthetic agents must be used for those undergoing ear surgery. In their research, Ozturk et al. demonstrated that the use of TIVA is safer, when compared to sevoflurane [6].

When anesthesia with a lower fresh gas flow is implemented, there is a cost reduction, prevention of environmental pollution, increased gas humidity, minimization of heat loss, and better preservation of the tracheal and bronchial physiology. Closer monitoring of the patients allows earlier realization of likely complications, and, thus, a safer anesthetic method is achieved through low-flow anesthesia [7, 8]. The pressure values of the patients who received low-flow anesthesia in our study followed an intraoperative lower middle ear pressure course, similar to the TIVA group when compared to the high-flow anesthesia.

We believe that low-flow anesthesia and TIVA implementations, which are advantageous in terms of anesthesia costs, environmental effects, and the health of the personnel, can be used more safely in middle ear surgeries, provided that a well-equipped anesthetic device and appropriate monitoring conditions are available.

## Figures and Tables

**Table 1:** Demographical and clinical features.

	Group I (*n* = 30)	Group II (*n* = 30)	Group III (*n* = 30)	*p*-value
Age (years)	47.0 ± 12.4	48.3 ± 10.1	49.3 ± 13.5	0.774^†^
Gender 0/1	12/18	13/17	12/18	0.955^‡^
Weight (kg)	79.9 ± 17.9	75.9 ± 13.3	73.9 ± 14.2	0.313^†^
Height (cm)	166.2 ± 9.9	168.5 ± 9.2	167.5 ± 7.2	0.583^†^
Body mass index (kg/m^2^)	28.8 ± 5.5	26.7 ± 3.9	26.2 ± 4.1	0.067^†^
ASA 1/2	14/16^a^	24/6^a^	21/9	**0.020** ^‡^
Duration of surgery (min)	70 (45–135)	65 (27–120)	71 (45–155)	0.659^¶^
Duration of anesthesia (min)	80 (60–150)	85 (45–145)	85 (58–175)	0.970^¶^

^†^One-way ANOVA, ^‡^Pearson's Chi-square, ^¶^Kruskal-Wallis test, and ^a^Group I versus Group II (*p* = 0.007).

**Table 2:** Repeated measurements of ear pressures (da Pa).

Time	Group I (*n* = 30)	Group II (*n* = 30)	Group III (*n* = 30)	*p* value^†^
*t*_1_	−37.4 ± 107.5	−45.5 ± 127.5	−72.9 ± 104.1	0.450
*t*_2_	−2.1 ± 91.4^a,b^	−114.6 ± 114.8^a^	−99.8 ± 99.4^b^	**<0.001**
*t*_3_	−31.7 ± 115.0	−95.1 ± 104.0	−106.0 ± 99.8	0.017
*t*_4_	−93.2 ± 93.7	−151.9 ± 87.1	−111.4 ± 101.9	0.053
*t*_5_	−142.0 ± 92.2	−108.2 ± 139.3	−117.2 ± 102.4	0.489
*t*_6_	−153.9 ± 88.6	−157.1 ± 81.3	−145.2 ± 96.5	0.865
*t*_7_	−126.7 ± 103.8	−138.0 ± 86.5	−143.2 ± 78.6	0.769

^†^One-way ANOVA; according to the Bonferroni Correction *p* < 0.0071 was considered as statistically significant, ^a^Group I versus Group II (*p* < 0.001). ^b^Group I versus Group III (*p* < 0.001).

**Table 3:** The differences in ear pressures regarding for baseline (*t*_1_).

Time	Group I (*n* = 30)	Group II (*n* = 30)	Group III (*n* = 30)	*p* value^†^
*t*_2_ − *t*_1_	−216.7 ± 613.0	99.1 ± 371.8	2.2 ± 392.3	0.054
*t*_3_ − *t*_1_	−65.1 ± 209.3	16.6 ± 249.3	−72.7 ± 172.6	0.814
*t*_4_ − *t*_1_	−119.9 ± 210.6	42.5 ± 406.0	−24.1 ± 300.5	0.164
*t*_5_ − *t*_1_	100.5 ± 1007.9	−122.5 ± 530.2	−31.7 ± 330.1	0.968
*t*_6_ − *t*_1_	238.4 ± 2037.7	−8.8 ± 515.7	32.6 ± 469.2	0.472
*t*_7_ − *t*_1_	−211.2 ± 705.0	3.4 ± 411.0	−11.7 ± 302.0	0.316

^†^One-way ANOVA; according to the Bonferroni Correction *p* < 0.0024 was considered as statistically significant.

**Table 4:** The differences in ear pressures regarding for *t*_3_.

Time	Group I (*n* = 30)	Group II (*n* = 30)	Group III (*n* = 30)	*p* value^†^
*t*_4_ − *t*_3_	−85.0 ± 164.6	−409.4 ± 1154.9	151.9 ± 599.3	0.159
*t*_5_ − *t*_3_	−56.9 ± 357.3	−332.7 ± 923.7	132.1 ± 636.5	0.205
*t*_6_ − *t*_3_	−24.7 ± 581.5	−345.2 ± 1089.2	162.3 ± 641.2	0.287
*t*_7_ − *t*_3_	−154.6 ± 349.1	−296.9 ± 1028.6	131.1 ± 575.7	0.036

^†^One-way ANOVA; according to the Bonferroni Correction *p* < 0.0024 was considered as statistically significant.
